# *catena*-Poly[[bis­(di­aqua­lithium)]-μ_4_-3,3′,5,5′-tetra­nitro-4,4′-bi­pyrazole-1,1′-diido]: a new moisture-insensitive alkali-metal energetic salt with a well-defined network structure

**DOI:** 10.1107/S2056989023005339

**Published:** 2023-06-20

**Authors:** Kostiantyn V. Domasevitch, Ganna A. Senchyk, Harald Krautscheid

**Affiliations:** a Inorganic Chemistry Department, National Taras Shevchenko University of Kyiv, Volodymyrska Str. 64/13, 01601 Kyiv, Ukraine; b Institute of Inorganic Chemistry, Leipzig University, Johannisallee 29, D-04103 Leipzig, Germany; University of Kentucky, USA

**Keywords:** crystal structure, lithium, nitro­pyrazoles, energetic materials, hydrogen bonding

## Abstract

In the structure of the title salt, [Li_2_(C_6_N_8_O_8_)(H_2_O)_4_]_*n*_, the 3,3′,5,5′-tetra­nitro-4,4′-bi­pyrazole-1,1′-diide dianion [{TNBPz}^2−^] is situated across the twofold axis. The distorted coordination octa­hedra around Li^+^ involve four short bonds with two pyrazolate N atoms and two aqua ligands [Li—N(O) = 1.999 (3)–2.090 (2) Å] and two longer contacts with nitro-O atoms [2.550 (2), 2.636 (2) Å]. When combined with μ_4_-{TNBPz}^2−^, this generates a mono-periodic polymeric structure incorporating discrete centrosymmeric [(H_2_O)_2_Li–(di­nitro­pyrazolato)_2_–Li(H_2_O)_2_] units. The three-dimensional stack of mutually orthogonal coordination chains is reminiscent of a Lincoln log pattern. It is influenced by conventional hydrogen bonding [O⋯O = 2.8555 (17)–3.0010 (15) Å] and multiple lone pair–π hole inter­actions of the nitro groups [N⋯O = 3.0349 (15) and 3.0887 (15) Å]. The Hirshfeld surface and two-dimensional fingerprint plots also support the significance of non-covalent bonding. Coordinative saturation and a favorable geometry at the Li^+^ ions, dense packing of the polymeric subconnectivities and particularly extensive inter­anion inter­actions may be involved in the stabilization of the structure. The title salt is a rare example of an energetic Li nitro­azolate, which nicely crystallizes from aqueous solution and is neither hygroscopic nor efflorescent. The TG/DTA data reveal total dehydration in the range of 330–430 K and stability of the anhydrous material up to 633–653 K.

## Abstract 2

In the title salt, the 3,3′,5,5′-tetra­nitro-4,4′-bi­pyrazole-1,1′-diide dianion [{TNBPz}^2−^] is situated across the twofold axis. The distorted coordination octa­hedra around Li^+^ involve four short bonds with two pyrazolate N atoms and two aqua ligands and two longer contacts with nitro-O atoms. When combined with μ_4_-{TNBPz}^2−^, this generates a mono-periodic polymeric structure incorporating discrete centrosymmeric [(H_2_O)_2_Li–(di­nitro­pyrazolato)_2_–Li(H_2_O)_2_] units. The three-dimensional stack of mutually orthogonal coordination chains is reminiscent of a Lincoln log pattern.

## 1. Chemical context

Red-light-emitting technical or military pyrotechnics trad­itionally concern the utilization of Sr salts (Sabatini, 2018 ▸). However, there is a growing inter­est for alternative red-flame colorants since strontium is potentially harmful to human health, specifically replacing calcium in bone and affecting skeletal development (Glück *et al.*, 2017 ▸). Recent works by Klapötke suggest significant potential for lithium-based systems, in particular those incorporating energetic nitro­pyrazole species (Dufter-Münster *et al.*, 2022 ▸; Dufter *et al.*, 2020 ▸). The accumulation of nitro groups enhances acidity (p*K*_a_ = 3.14 for 3,5-di­nitro­pyrazole *vs* 14.63 for the parent pyrazole; Janssen *et al.*, 1973 ▸) for producing hydrolytically stable salts, while the incorporation of energy-rich nitro­pyrazolates contributes to oxygen balance of the formulations. In addition, the high nitro­gen content and N—N linkage within the pyrazole ring inherently facilitate the release of nitro­gen gas when burned. This meets the needs for cooling the flame for improving the color purity (Glück *et al.*, 2017 ▸).

However, most of the examined salts are still not suited for applications in spite of such valuable pre-requisites. The nitro­pyrazolates crystallize with difficulty (Drukenmüller *et al.*, 2014 ▸) and their Li salts are commonly hygroscopic and deliquescent (Dufter-Münster *et al.*, 2022 ▸). In the present work, we address this problem with a crystal-engineering approach. The recently introduced bifunctional tecton 3,3′,5,5′-tetra­nitro-4,4′-bi­pyrazole [H_2_(TNBPz)] readily affords a range of salts with alkali metal ions (Domasevitch & Ponomarova, 2021 ▸) and nitro­gen bases (Gospodinov *et al.*, 2020 ▸) and supports either coordination or hydrogen-bonded arrays in a very predictable fashion. One can anti­cipate that the doubled nitro­pyrazolate functionality could grant the connection of the Li^+^ ions and generation of a relatively robust polymer, whereas the extended mol­ecular framework of {TNBPz}^2−^ is particularly beneficial for the dense anion–anion inter­actions because of a larger contribution of dispersion forces. An appropriate set of binding sites for such inter­actions may be found with four NO_2_ functions, which commonly act as self-complementary donor and acceptor groups for non-covalent lone pair–π hole bonds (Bauzá *et al.*, 2017 ▸). With this in mind, we prepared the new energetic salt *catena*-poly[[bis­(di­aqua­lithium)]-μ_4_-3,3′,5,5′-tetra­nitro-4,4′-bi­pyrazole-1,1′-diido] and report its structure here. 
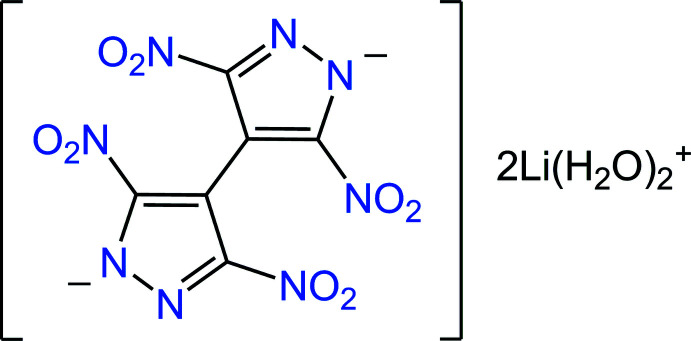


## 2. Structural commentary

**Figure 1:**
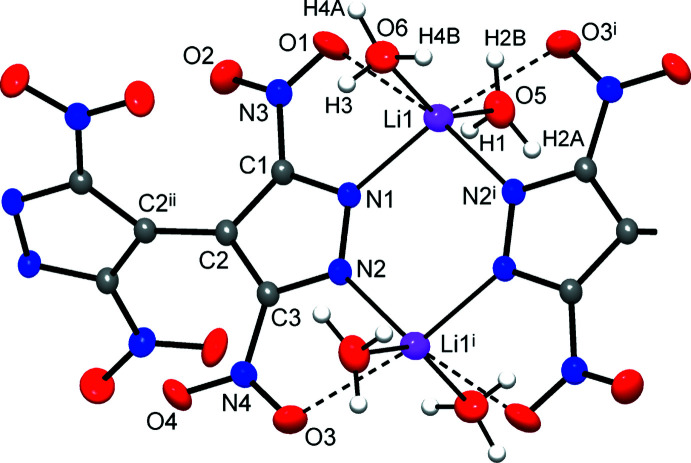
The mol­ecular structure of the title compound with displacement ellipsoids drawn at the 50% probability level. Dotted lines indicate distal Li—O(nitro) inter­actions. Two out of four H atoms of the aqua ligands are equally disordered over two positions (H2*A*, H2*B* and H4*A*, H4*B*). [Symmetry codes: (i) −*x* + 1, −*y* + 1, −*z*; (ii) −*x* + 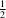 , −*y* + 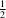 , *z*.]

The mol­ecular structure of the title compound is shown in Fig. 1 ▸, with the asymmetric unit comprising one metal ion, two aqua ligands and half a mol­ecule of the organic dianion {TNBPz}^2−^, which is situated across the twofold axis passing through the center of the C—C bond between two pyrazole rings.

The coordination around the Li^+^ ion may be regarded as distorted octa­hedral, with four relatively short bonds with two pyrazole-N atoms [2.086 (2) and 2.090 (2) Å], two aqua-O atoms [1.999 (3) and 2.027 (3) Å] and two elongated bonds with nitro-O atoms [2.550 (2) and 2.636 (2) Å] (Table 1 ▸). A very comparable pattern for lithium 4-amino-3,5-di­nitro­pyrazolate retained only one Li—O(nitro) bond, which was slightly shorter [2.441 (4) Å; Dufter-Münster *et al.*, 2022 ▸]. Although the distorted octa­hedral geometries themselves are well known for Li^+^ (Olsher *et al.*, 1991 ▸), the spread of the bond lengths is usually narrower. For example, the citrate salt exhibits six Li—O bonds in the range of 1.998 (2)–2.222 (3) Å (Rossi *et al.*, 1983 ▸). The exceedingly long bonds with nitro groups may be described rather as very weak ion-dipole contacts, while the remaining four shorter bonds almost perfectly match the sum of the corresponding ionic radii for 4-coordinate Li^+^ ions [which are Li—O = 1.94 Å and Li—N = 2.05 Å; Shannon, 1976 ▸]. Nevertheless, the weak Li—O(nitro) inter­actions are presumably important for a more effective shielding of the cations against hydration when exposed to moist air. Unlike many related systems, crystals of the title compound are not hygroscopic. A second issue is the saturation of the Li^+^ environment with an appropriate number of aqua ligands. This is contrary to the structures of far more moisture-sensitive 3,4-, 3,5-di­nitro­pyrazolates and 4-amino-3,5-di­nitro­pyrazolate, where the di­aqua­lithium moieties were recognized as local fragments of 1:1 aqua­lithium chains –Li–[(μ-H_2_O)Li]_*n*_– (Dufter-Münster *et al.*, 2022 ▸).

The geometric parameters of the {TNBPz}^2−^ dianion are consistent with the data for neutral H_2_(TNBPz) (Domasevitch *et al.*, 2019 ▸) or singly charged species {H(TNBPz)}^−^ (Domasevitch & Ponomarova, 2021 ▸). The ion adopts a twisted conformation with two pyrazole rings rotated by 54.48 (4)° (Fig. 1 ▸). Although this twist is larger than 42.99 (8)° for Rb{H(TNBPz)} (Domasevitch & Ponomarova, 2021 ▸), it is still unusually small, as may be compared with the even less hindered 3,3′,5,5′-tetra­methyl-4,4′-bi­pyrazole analogs (65–90°; Ponomarova *et al.*, 2013 ▸). In fact, the intra­molecular inter­actions of NO_2_ groups, with a shortest contact N3⋯O4^ii^ = 2.9199 (15) Å [symmetry code: (ii) −*x* + 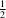 , −*y* + 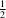 , *z*], are likely attractive, as a kind of lone pair–π hole bonding (Bauzá *et al.*, 2017 ▸). The central C2—C2^ii^ bond is insensitive to the protolytic effects [1.463 (2) Å *vs* 1.4644 (15) and 1.462 (2) Å for H_2_(TNBPz) and Rb{H(TNBPz)}, respectively], which indicates a lack of essential conjugation between two pyrazolate rings. Shortening of the C-NO_2_ bonds upon deprotonation is also minor [mean 1.4308 (16) Å *vs* 1.439 (2) Å for H_2_(TNBPz)], while a certain increase in conjugation is reflected rather by a perceptible flattening of the di­nitro­pyrazole fragments. For the latter, the NO_2_ groups are nearly coplanar with the ring, the two dihedral angles are 1.35 (8) and 11.66 (8)°, for N4O3O4 and N3O1O2 groups, respectively. In the case of H_2_(TNBPz), the twist comes to 22.8 (2)°. The most appreciable consequence of the dianionic structure is similarity of bond angles at the ring-N atoms: N2—N1—C1 = 107.11 (9)° and N1—N2—C3 = 106.93 (9)°. For the neutral di­nitro­pyrazole rings, the parameters for N- [103.9 (2)°] and NH-sites [112.0 (2)°] are clearly different (Domasevitch *et al.*, 2019 ▸).

## 3. Supra­molecular features

**Figure 2:**
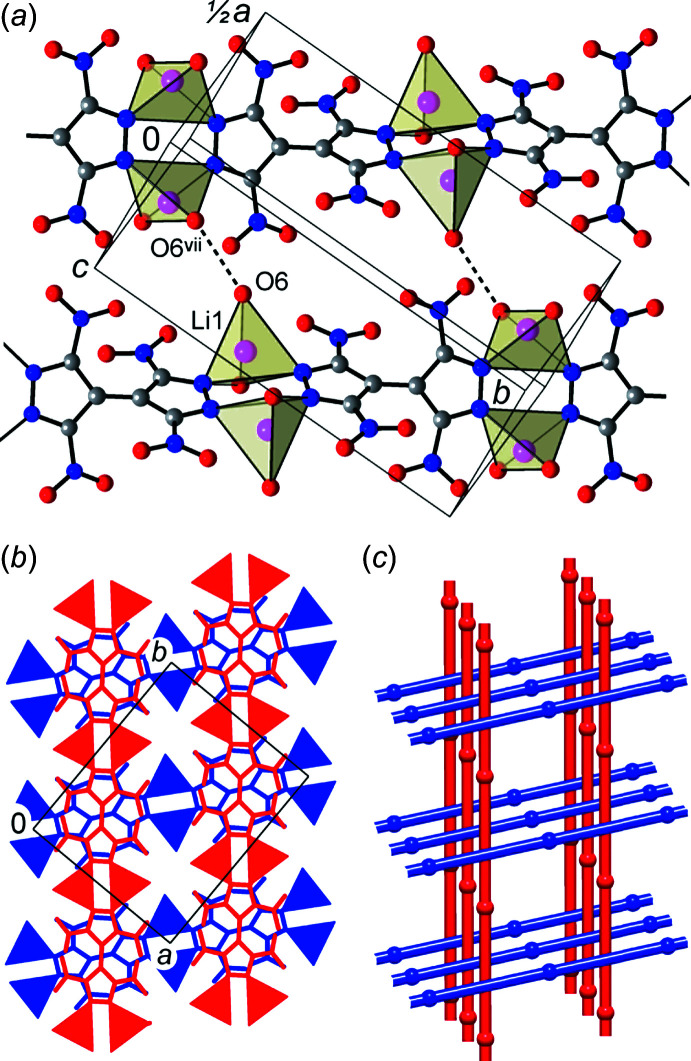
(*a*) View of the coordination chains with hydrogen-bond inter­actions between pairs of symmetry-related aqua ligands that afford layers parallel to the *ab* plane. The Li^+^ ions are represented by coordination tetra­hedra, while considering only the four shortest coordination bonds. (*b*) Projection of the structure on the *ab* plane with two successive layers indicated in blue and red. (*c*) Packing of the coordination chains following a Lincoln log pattern. The chain nodes represent the dilithium units and the chain links are bridging {TNBPz}^2−^ anions. [Symmetry code: (vii) −*x* + 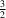 , −*y* + 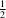 , *z*.]

The title compound adopts a mono-periodic polymeric structure with the {TNBPz}^2−^ anions acting as tetra­dentate bridging ligands. Two di­nitro­pyrazolate groups of the anions and two di­aqua­lithium fragments compose the cyclic pattern (Fig. 2 ▸), which is reminiscent of the dimers in lithium 3,5-di­nitro-4-amino- and 3,4-di­nitro­pyrazolates (Dufter-Münster *et al.*, 2022 ▸). Unlike these monofunctional prototypes, with the C2—C2^ii^ bond linking the two pz halves of the bi­pyrazole core, these dimers are connected into linear chains, with a distance of 9.06 Å between the centroids of the Li_2_(pz)_2_ cycles (pz is pyrazole).

**Figure 3:**
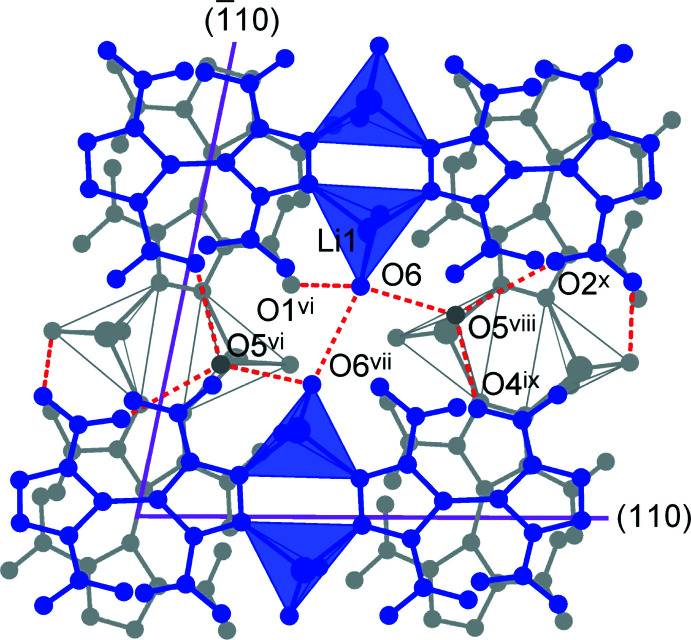
The hydrogen bonding between adjacent coordination chains, which is shown by the dotted red lines. Two successive layers are marked in blue and gray and the Li^+^ ions are presented as coodination tetra­hedra, while considering only the four shortest coordination bonds for clarity. The purple lines identify the directions of the coordination chains, which coincide with the crystal directions (110) and ( 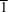 10). [Symmetry codes: (vi) *x*, −*y* + 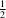 , *z* − 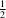 ; (vii) −*x* + 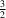 , −*y* + 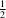 , *z*; (ix) *x* + 1, *y*, *z*; (*x*) *x* + 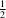 , *y* + 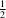 , −*z*.]

Adjacent chains are linked by a set of conventional hydrogen bonds O—H⋯O, which involve either aqua or nitro-O acceptors. The geometric parameters for five types of such inter­actions are very comparable (Table 2 ▸), with the range of O⋯O separations [2.8555 (17)–3.0010 (15) Å] and nearly straight angles at the H atoms [153 (2)–177 (4)°] indicating directional hydrogen bonding. Bonds of the type O6—H4*A*⋯O6^vii^ [symmetry code: (vii) −*x* + 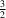 , −*y* + 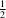 , *z*; the H atom is equally disordered over two symmetry-related O atoms] are important for the connection of the chains into layers, which are parallel to the *ab* plane, whereas the second aqua/aqua bond O5—H⋯O6^v^ [symmetry code: (v) −*x* + 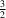 , *y*, *z* + 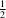 ] and all three aqua/nitro hydrogen bonds actualize between the layers (Fig. 3 ▸).

**Figure 4:**
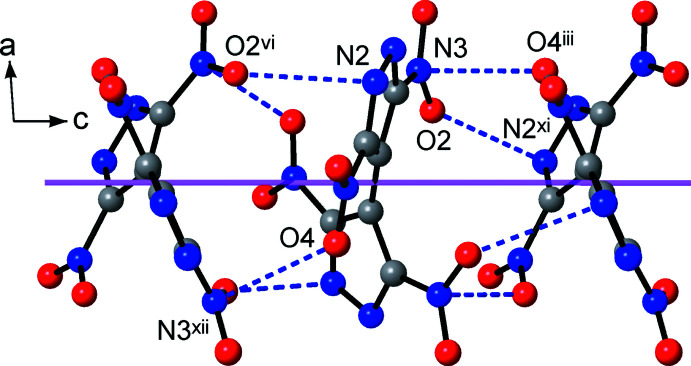
Lone pair-π–hole inter­actions of the {TNBPz}^2−^ anions. The stacking axis is indicated by the purple line and it coincides with the *c*-axis direction. [Symmetry codes: (iii) −*x* + 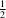 , *y*, *z* + 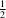 ; (vi) *x*, −*y* + 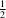 , *z* − 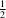 ; (xi) *x*, −*y* + 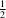 , *z* + 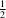 ; (xii) −*x* + 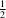 , *y*, *z* − 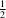 .]

The coordination chains of two successive layers are inclined, one in relation to the other, and adopt an angle of 78.9° [which is the angle between the (110) and ( 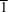 10) directions in the crystal]. This nearly orthogonal mutual orientation conditions a very simple packing pattern, in the form of Lincoln log-like stacks (Fig. 2 ▸). The {TNBPz}^2−^ anions are situated exactly one on the top of the other at the distances of 4.71 Å corresponding to one half of the *c* parameter of the unit cell. In spite of the twisted conformation of the bi­pyrazole, such stacking is geometrically favorable, with every pair of mol­ecules within the stack mutually fitting like puzzles. The resulting inter­actions are particularly extensive, with four pairs of symmetry-related short contacts N3⋯O4^iii^ = 3.0349 (15) Å and N2⋯O2^vi^ = 3.0887 (15) Å [symmetry codes: (iii) −*x* + 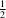 , *y*, *z* + 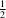 ; (vi) *x*, −*y* + 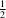 , *z* − 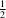 ] established by every {TNBPz}^2−^ anion (Fig. 4 ▸). For the mutually bonded nitro groups, *i.e.* N3O1O2 and (N4O3O4)^iii^, the latter is lone-pair donor and the former one is π-hole acceptor, which combine to create a very characteristic stack (Veluthaparambath *et al.*, 2023 ▸). The planes of the two groups subtend a dihedral angle of 34.16 (15)°, but the N3⋯O4^iii^ axis is nearly orthogonal to the acceptor plane, as indicated by a slippage angle of 8.6 (2)°. The second type of inter­action of is a lone pair–π-hole bond with the di­nitro­pyrazolate ring system. Similar inter­actions are well known for electron-deficient N-heterocycles and they are most pronounced for 1,2,4,5-tetra­zines (Gural’skiy *et al.*, 2009 ▸). In this case, the inter­planar [49.96 (10)°] and slippage angles [13.98 (15)°, with respect to the centroid of the pyrazole ring] are slightly larger. This non-covalent bonding is clearly traced in every structure adopted by H_2_(TNBPz) [with very short mutual nitro contacts down to N⋯O = 2.9115 (15) Å; Domasevitch *et al.*, 2019 ▸] and its anions (Domasevitch & Ponomarova, 2021 ▸) and in fact it may be regarded as a prominent feature for the crystal chemistry of such systems. These close inter­actions of shape-complementary twisted mol­ecules contribute to the relatively high packing index of 75.8%, which is at the top of the 65–75% range expected for organic solids (Dunitz, 1995 ▸).

## 4. Hirshfeld analysis

**Figure 5:**
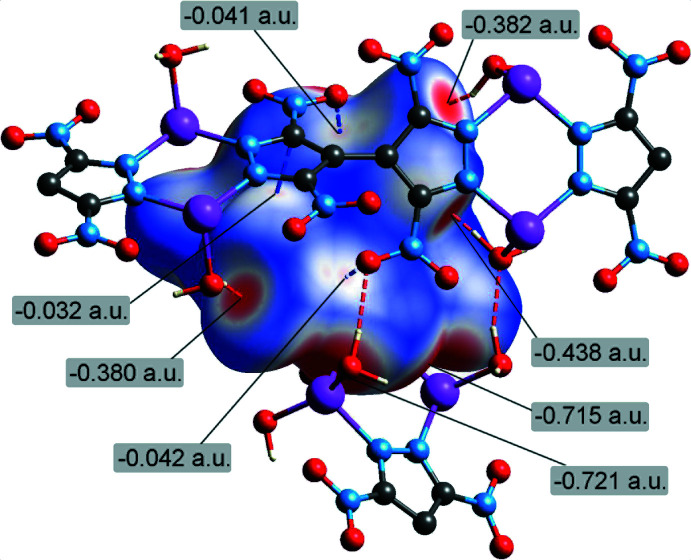
Hirshfeld surface of the individual {TNBPz}^2−^ anion, mapped over *d*_norm_ (the C—H distances are normalized) in the color range −0.73 (red) to 1.14 a.u. (blue), with the red regions indicating the sites of inter­molecular inter­actions.

The supra­molecular inter­actions in the title structure were further assessed by Hirshfeld surface analysis (Spackman & Byrom, 1997 ▸; McKinnon *et al.*, 2004 ▸; Hirshfeld, 1977 ▸; Spackman & McKinnon, 2002 ▸) performed with *CrystalExplorer17* (Turner *et al.*, 2017 ▸). The Hirshfeld surface of the individual {TNBPz}^2−^ anion mapped over *d*_norm_, using a fixed color scale of −0.73 (red) to 1.14 a.u. (blue), indicates a set of red spots associated with the inter­action sites (Fig. 5 ▸). Two pairs of the most intense spots (−0.72 a.u.) are associated with the Li—N coordination, while the hydrogen bonding is also visualized as prominent features (−0.38 to −0.44 a.u.). The lone pair–π-hole inter­actions of NO_2_ groups are less visible, but they are still detectable on the surface as a set of very diffuse spots (−0.04 a.u.).

**Figure 6:**
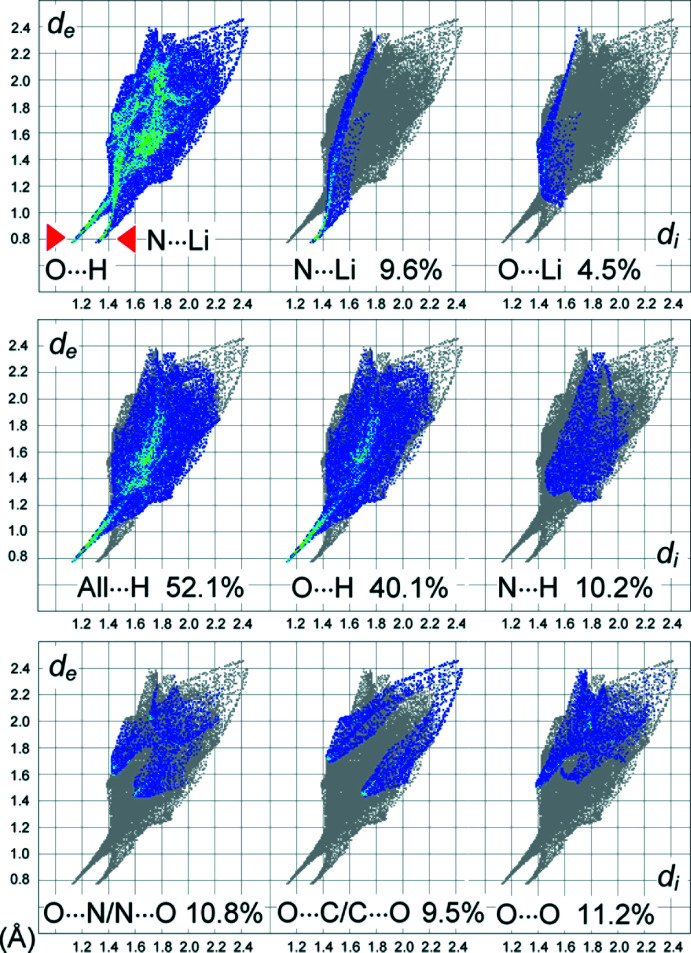
Two-dimensional fingerprint plots for the individual {TNBPz}^2−^ anion, and delineated into the principal contributions of N⋯Li, O⋯Li, O⋯H, N⋯H, O⋯N/N⋯O, O⋯C/C⋯O and O⋯O contacts. Other contributors are: C⋯N/N⋯C, 1.8%; N⋯N, 0.5% and C⋯C, 0.1% contacts.

The two-dimensional fingerprint plots (Fig. 6 ▸) are even more informative. They suggest the significance of coordination and hydrogen-bonding inter­actions, which are reflected as two sharp spikes pointing to the lower left with the shortest contacts N⋯Li = 2.1 Å and O⋯H = 2.0 Å. One can note a similar indication of N⋯Li (9.6%) and O⋯Li (4.5%) contacts, but the fraction of the latter is significantly less and the corresponding short spike is diffuse. This agrees with the weakness of the coordination bonds adopted by the nitro-O atoms. O⋯H inter­actions account for 40.1% of the entire number of contacts. This is complemented by a 10.2% contribution of N⋯H contacts, but there are no signs of any O—H⋯N bonding. The plot represents a rather diffuse collection of points with the shortest N⋯H = 2.8 Å. The large fraction of O⋯N/N⋯O and O⋯C/C⋯O contacts (in total over 20%) is a primary indicator for extensive anion–anion inter­actions. The nature of these contacts is similar and they appear in the plots as nearly symmetrical (about the diagonal where *d*_i_ = *d*_e_) pairs of features. Therefore, either donor or acceptor sites of the bonds are found within the individual anions supporting the shortest contacts O⋯N = 3.0 Å and O⋯C = 3.1 Å. It may be postulated that the accessibility of aqua hydrogen-bond donors does not disrupt the main anion–anion inter­actions, but rather governs elimination of less favorable nitro O⋯O contacts. The total contributions of the C(N)⋯C(N,O) contacts in the title structure and in the very similar unsolvated Rb{H(TNBPz)} (Domasevitch & Ponomarova, 2021 ▸) are nearly identical (33.4% and 31.9%, respectively), while the impact of hydrogen bonding is best illustrated by the pronounced contraction of the O⋯O fraction (37.4% to 11.2% in the present case). Moreover, the asymmetry of the O⋯O plot is contrary to the patterns for the O⋯N/N⋯O and O⋯C/C⋯O contacts. This witnesses the prevalence of the nitro/aqua contacts, instead of direct nitro O⋯O inter­actions.

## 5. Synthesis and crystallization

3,3′,5,5′-Tetra­nitro-4,4′-bi­pyrazole monohydrate [H_2_(TNBPz)·H_2_O] is readily available by nitration of 4,4′-bi­pyrazole in mixed acids (yield 92%) and subsequent crystallization from water (Domasevitch *et al.*, 2019 ▸).

For the preparation of the title compound, 0.294 g (7.0 mmol) of LiOH·H_2_O was dissolved in 10 ml of water at 333–343 K and then 1.162 g (3.5 mmol) of solid H_2_(TNBPz)·H_2_O was added with stirring. The mixture was stirred for 30 min and the resulting clear deep-yellow solution was cooled to r.t. Slow evaporation to a minimum volume over 8–10 d led to crystallization of the product as well-developed large lemon-yellow prisms. The crystals were removed and dried on a filter paper in air. The yield was 1.25 g (90%). The material shows neither signs of hygroscopy nor efflorescence when exposed to ambient air for months.

Analysis (%) calculated for C_6_H_8_Li_2_N_8_O_12_: C 18.10, H 2.03, N 28.15; found: C 18.45, H 1.99, N 28.42. IR (KBr, cm^−1^): 564 *m*, 636 *m*, 698 *m*, 716 *m*, 773 *w*, 853 *s*, 1006 *m*, 1021 *s*, 1189 *w*, 1284 *w*, 1308 *s*, 1321 *s*, 1353 *s*, 1381 *s*, 1410 *s*, 1484 *s*, 1540 *s*, 1641 *m*, 1658 *m*, 3460 *br*, 3580 *br*.

The FT–IR spectrum reveals a distinctive pattern, which is characteristic for hydrated nitro­pyrazolates. The peaks, which are associated with the aqua ligands, appear at 3460 and 3580 cm^−1^ (O—H stretching), 1641, 1658 cm^−1^ (bend) and 564 cm^−1^ (libration). The peaks for symmetric and asymmetric NO_2_ stretching (1351, 1381 and 1484, 1540 cm^−1^, respectively) are very similar to the spectra of comparable 3,5-di­nitro­pyrazole (Ravi, 2015 ▸). These double peaks originate in coupling of the NO_2_ vibrations with the ring motions. The intense and sharp band at 853 cm^−1^ is ν(C—NO_2_), and its shift, with respect to the band for H_2_(TNBPz)·H_2_O (839 cm^−1^; Domasevitch *et al.*, 2019 ▸), suggests a certain increase of conjugation of the nitro groups with the carrier aromatic ring upon deprotonation. For Rb{H(TNBPz)}, both these frequencies were present (839 and 852 cm^−1^; Domasevitch & Ponomarova, 2021 ▸).

**Figure 7:**
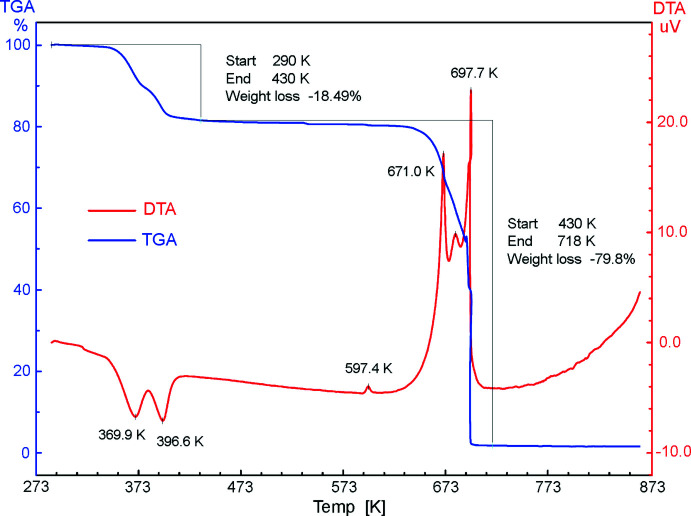
Combined DTA (red) and TGA (blue) plots for the title compound, in the temperature range of 273–873 K (air, heating rate 5 K min^−1^).

Preliminary assays for safety of the title compound and its suitability for pyrotechnic formulations were performed by thermal analysis (OZM Research DTA 552-Ex). There are two partially separated stages for nearly identical weight losses in the temperature range of 330–430 K (Fig. 7 ▸), which correspond to total dehydration of the salt (in total, −18.49 mass %; −4H_2_O: calculated −18.09%). The anhydrous material is stable up to 633 K, with the very minor exothermic event at 597 K possibly indicating a phase transition. Exothermic decomposition proceeds above 653 K, with instantaneous loss of any remaining weight and a sharp exothermic effect at *ca* 700 K suggesting an explosion. For comparison, typical onset temperatures for decomposition of energetic Li nitro­pyrazolates are 400–500 K, and only 3,5-di­nitro­pyrazolate is stable up to 600 K (Dufter-Münster *et al.*, 2022 ▸).

## 6. Refinement

Crystal data, data collection and structure refinement details are summarized in Table 3 ▸. The hydrogen atoms were located and then refined with isotropic thermal parameters. For both aqua ligands, one of the H atoms is equally disordered over two positions, which were refined with 0.5 partial contribution factors and with soft similarity restraints applied to O—H bond lengths [O—H = 0.82 (3)–0.95 (5) Å].

## Supplementary Material

Crystal structure: contains datablock(s) global, I. DOI: 10.1107/S2056989023005339/pk2688sup1.cif

Structure factors: contains datablock(s) I. DOI: 10.1107/S2056989023005339/pk2688Isup2.hkl

CCDC reference: 2269963

Additional supporting information:  crystallographic information; 3D view; checkCIF report

## Figures and Tables

**Table 1:** Selected geometric parameters (Å, °)

Li1—O5	1.999 (3)	Li1—N2^i^	2.090 (2)
Li1—O6	2.027 (3)	Li1—O3^i^	2.550 (2)
Li1—N1	2.086 (2)	Li1—O1	2.636 (2)
			
O5—Li1—O6	145.63 (13)	N1—Li1—O3^i^	170.20 (11)
O5—Li1—N1	98.49 (10)	N2^i^—Li1—O3^i^	68.60 (7)
O6—Li1—N1	101.69 (11)	O5—Li1—O1	80.47 (8)
O5—Li1—N2^i^	100.84 (11)	O6—Li1—O1	81.94 (9)
O6—Li1—N2^i^	101.22 (11)	N1—Li1—O1	67.50 (7)
N1—Li1—N2^i^	103.29 (10)	N2^i^—Li1—O1	170.76 (11)
O5—Li1—O3^i^	78.28 (8)	O3^i^—Li1—O1	120.49 (9)
O6—Li1—O3^i^	85.66 (8)		

Symmetry code: (i) 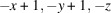 .

**Table 2:** Hydrogen-bond geometry (Å, °)

*D*—H⋯*A*	*D*—H	H⋯*A*	*D*⋯*A*	*D*—H⋯*A*
O5—H1⋯O4^ii^	0.87 (2)	2.02 (2)	2.8818 (14)	174 (2)
O5—H2*A*⋯O2^iii^	0.87 (2)	2.13 (2)	3.0010 (15)	177 (4)
O5—H2*B*⋯O6^iv^	0.87 (2)	2.02 (2)	2.8555 (17)	163 (3)
O6—H3⋯O1^v^	0.82 (3)	2.24 (3)	2.9995 (17)	153 (2)
O6—H4*A*⋯O6^vi^	0.92 (4)	2.01 (4)	2.905 (2)	163 (4)
O6—H4*B*⋯O5^vii^	0.95 (5)	1.93 (5)	2.8555 (17)	164 (4)

Symmetry codes: (ii) 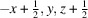 ; (iii) 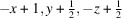 ; (iv) 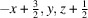 ; (v) 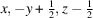 ; (vi) 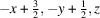 ; (vii) 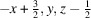 .

**Table 3:** Experimental details

Crystal data	
Chemical formula	[Li_2_(C_6_N_8_O_8_)(H_2_O)_4_]
*M*_r_	398.08
Crystal system, space group	Orthorhombic, *P* *c* *c* *n*
Temperature (K)	213
*a*, *b*, *c* (Å)	11.5094 (9), 13.9839 (8), 9.4247 (5)
*V* (Å^3^)	1516.87 (17)
*Z*	4
Radiation type	Mo *K*α
μ (mm^−1^)	0.17
Crystal size (mm)	0.25 × 0.22 × 0.20

Data collection	
Diffractometer	Stoe Image plate diffraction system
No. of measured, independent and observed [*I* > 2σ(*I*)] reflections	10855, 1817, 1225
*R*_int_	0.049
(sin θ/λ)_max_ (Å^−1^)	0.660

Refinement	
*R*[*F* ^2^ > 2σ(*F* ^2^)], *wR*(*F* ^2^), *S*	0.030, 0.072, 0.88
No. of reflections	1817
No. of parameters	151
No. of restraints	4
H-atom treatment	All H-atom parameters refined
Δρ_max_, Δρ_min_ (e Å^−3^)	0.28, −0.14

Computer programs: *IPDS Software* (Stoe & Cie, 2000 ▸), *SHELXS97* (Sheldrick, 2008 ▸), *SHELXL2014/7* (Sheldrick, 2015 ▸), Diamond (Brandenburg, 1999 ▸) and *WinGX* (Farrugia, 2012 ▸).

## supplementary crystallographic information

### Crystal data

**Table:**

[Li_2_(C_6_N_8_O_8_)(H_2_O)_4_]	*D*_x_ = 1.743 Mg m^−^^3^
*M**_r_* = 398.08	Mo *K*α radiation, λ = 0.71073 Å
Orthorhombic, *P**c**c**n*	Cell parameters from 8000 reflections
*a* = 11.5094 (9) Å	θ = 2.9–28.0°
*b* = 13.9839 (8) Å	µ = 0.17 mm^−^^1^
*c* = 9.4247 (5) Å	*T* = 213 K
*V* = 1516.87 (17) Å^3^	Prism, yellow
*Z* = 4	0.25 × 0.22 × 0.20 mm
*F*(000) = 808	

### Data collection

**Table:**

Stoe Image plate diffraction system diffractometer	*R*_int_ = 0.049
φ oscillation scans	θ_max_ = 28.0°, θ_min_ = 2.9°
10855 measured reflections	*h* = −15→15
1817 independent reflections	*k* = −16→18
1225 reflections with *I* > 2σ(*I*)	*l* = −12→11

### Refinement

**Table:**

Refinement on *F*^2^	Primary atom site location: structure-invariant direct methods
Least-squares matrix: full	Secondary atom site location: difference Fourier map
*R*[*F*^2^ > 2σ(*F*^2^)] = 0.030	Hydrogen site location: difference Fourier map
*wR*(*F*^2^) = 0.072	All H-atom parameters refined
*S* = 0.88	*w* = 1/[σ^2^(*F*_o_^2^) + (0.0455*P*)^2^] where *P* = (*F*_o_^2^ + 2*F*_c_^2^)/3
1817 reflections	(Δ/σ)_max_ < 0.001
151 parameters	Δρ_max_ = 0.28 e Å^−^^3^
4 restraints	Δρ_min_ = −0.14 e Å^−^^3^

### Special details

**Table:**

Geometry. All esds (except the esd in the dihedral angle between two l.s. planes) are estimated using the full covariance matrix. The cell esds are taken into account individually in the estimation of esds in distances, angles and torsion angles; correlations between esds in cell parameters are only used when they are defined by crystal symmetry. An approximate (isotropic) treatment of cell esds is used for estimating esds involving l.s. planes.

### Fractional atomic coordinates and isotropic or equivalent isotropic displacement parameters (Å^2^)

**Table:**

	*x*	*y*	*z*	*U*_iso_*/*U*_eq_	Occ. (<1)
Li1	0.62380 (18)	0.42289 (16)	0.0859 (2)	0.0258 (5)	
O1	0.57465 (8)	0.25173 (7)	0.18668 (12)	0.0401 (3)	
O2	0.42324 (9)	0.16320 (7)	0.21808 (12)	0.0357 (3)	
O3	0.17428 (9)	0.50551 (7)	−0.11938 (11)	0.0341 (3)	
O4	0.08500 (8)	0.37055 (7)	−0.08988 (11)	0.0327 (3)	
O5	0.62403 (9)	0.45230 (8)	0.29341 (11)	0.0336 (3)	
O6	0.69704 (11)	0.34427 (9)	−0.07111 (14)	0.0361 (3)	
N1	0.44846 (8)	0.38814 (7)	0.06362 (11)	0.0196 (2)	
N2	0.36602 (8)	0.43947 (7)	−0.00029 (12)	0.0191 (2)	
N3	0.47107 (9)	0.23391 (7)	0.16949 (12)	0.0236 (3)	
N4	0.17120 (9)	0.42244 (8)	−0.07765 (11)	0.0212 (2)	
C1	0.40259 (10)	0.30164 (9)	0.09198 (13)	0.0187 (3)	
C2	0.28824 (10)	0.29180 (9)	0.04627 (13)	0.0176 (3)	
C3	0.27171 (10)	0.38324 (8)	−0.01021 (13)	0.0178 (3)	
H1	0.5584 (14)	0.4317 (13)	0.327 (2)	0.071 (7)*	
H2A	0.608 (3)	0.5133 (13)	0.289 (4)	0.064 (13)*	0.5
H2B	0.671 (2)	0.409 (2)	0.326 (4)	0.047 (10)*	0.5
H3	0.651 (2)	0.3349 (17)	−0.136 (3)	0.089 (9)*	
H4A	0.724 (4)	0.284 (3)	−0.053 (4)	0.062 (13)*	0.5
H4B	0.749 (4)	0.390 (3)	−0.108 (5)	0.081 (15)*	0.5

### Atomic displacement parameters (Å^2^)

**Table:**

	*U*^11^	*U*^22^	*U*^33^	*U*^12^	*U*^13^	*U*^23^
Li1	0.0233 (10)	0.0256 (12)	0.0285 (13)	−0.0004 (9)	0.0005 (9)	0.0042 (10)
O1	0.0233 (5)	0.0431 (6)	0.0540 (7)	−0.0006 (4)	−0.0134 (5)	0.0113 (5)
O2	0.0439 (6)	0.0233 (5)	0.0397 (6)	−0.0057 (4)	−0.0055 (5)	0.0132 (4)
O3	0.0329 (5)	0.0264 (5)	0.0431 (7)	0.0014 (4)	−0.0059 (4)	0.0133 (5)
O4	0.0219 (5)	0.0358 (6)	0.0406 (6)	−0.0075 (4)	−0.0109 (4)	0.0059 (5)
O5	0.0371 (6)	0.0374 (7)	0.0264 (6)	−0.0090 (5)	−0.0017 (5)	0.0036 (5)
O6	0.0336 (6)	0.0380 (7)	0.0369 (7)	0.0057 (5)	−0.0007 (5)	−0.0095 (5)
N1	0.0195 (5)	0.0176 (5)	0.0218 (6)	−0.0027 (4)	−0.0024 (4)	0.0020 (4)
N2	0.0190 (5)	0.0186 (5)	0.0197 (5)	−0.0032 (4)	0.0003 (4)	0.0014 (4)
N3	0.0257 (6)	0.0221 (6)	0.0229 (6)	−0.0016 (4)	−0.0038 (5)	0.0016 (5)
N4	0.0207 (5)	0.0226 (6)	0.0204 (6)	−0.0010 (4)	−0.0013 (4)	0.0016 (5)
C1	0.0187 (6)	0.0178 (6)	0.0195 (6)	−0.0028 (5)	−0.0011 (5)	0.0014 (5)
C2	0.0184 (6)	0.0178 (6)	0.0167 (6)	−0.0026 (5)	0.0008 (5)	−0.0013 (5)
C3	0.0177 (6)	0.0183 (6)	0.0174 (6)	−0.0021 (5)	−0.0006 (5)	−0.0007 (5)

### Geometric parameters (Å, º)

**Table:**

Li1—O5	1.999 (3)	O5—H2B	0.866 (17)
Li1—O6	2.027 (3)	O6—H3	0.82 (3)
Li1—N1	2.086 (2)	O6—H4A	0.92 (4)
Li1—N2^i^	2.090 (2)	O6—H4B	0.95 (5)
Li1—O3^i^	2.550 (2)	N1—N2	1.3336 (14)
Li1—O1	2.636 (2)	N1—C1	1.3465 (16)
O1—N3	1.2287 (14)	N2—C3	1.3435 (15)
O2—N3	1.2209 (14)	N2—Li1^i^	2.090 (2)
O3—N4	1.2270 (14)	N3—C1	1.4324 (16)
O3—Li1^i^	2.550 (2)	N4—C3	1.4292 (16)
O4—N4	1.2346 (13)	C1—C2	1.3917 (16)
O5—H1	0.869 (15)	C2—C3	1.3981 (17)
O5—H2A	0.874 (17)	C2—C2^ii^	1.463 (2)
			
O5—Li1—O6	145.63 (13)	H3—O6—H4A	102 (3)
O5—Li1—N1	98.49 (10)	Li1—O6—H4B	99 (3)
O6—Li1—N1	101.69 (11)	H3—O6—H4B	105 (3)
O5—Li1—N2^i^	100.84 (11)	N2—N1—C1	107.11 (9)
O6—Li1—N2^i^	101.22 (11)	N2—N1—Li1	127.46 (10)
N1—Li1—N2^i^	103.29 (10)	C1—N1—Li1	124.64 (10)
O5—Li1—O3^i^	78.28 (8)	N1—N2—C3	106.93 (9)
O6—Li1—O3^i^	85.66 (8)	N1—N2—Li1^i^	129.06 (9)
N1—Li1—O3^i^	170.20 (11)	C3—N2—Li1^i^	123.87 (10)
N2^i^—Li1—O3^i^	68.60 (7)	O2—N3—O1	123.53 (11)
O5—Li1—O1	80.47 (8)	O2—N3—C1	118.60 (10)
O6—Li1—O1	81.94 (9)	O1—N3—C1	117.84 (10)
N1—Li1—O1	67.50 (7)	O3—N4—O4	123.35 (11)
N2^i^—Li1—O1	170.76 (11)	O3—N4—C3	118.83 (10)
O3^i^—Li1—O1	120.49 (9)	O4—N4—C3	117.81 (10)
N3—O1—Li1	110.17 (9)	N1—C1—C2	113.46 (11)
N4—O3—Li1^i^	111.00 (9)	N1—C1—N3	118.65 (10)
Li1—O5—H1	106.9 (14)	C2—C1—N3	127.80 (11)
Li1—O5—H2A	99 (3)	C1—C2—C3	98.98 (10)
H1—O5—H2A	99.0 (19)	C1—C2—C2^ii^	130.38 (13)
Li1—O5—H2B	102 (2)	C3—C2—C2^ii^	130.46 (12)
H1—O5—H2B	100.4 (19)	N2—C3—C2	113.51 (10)
Li1—O6—H3	111.1 (18)	N2—C3—N4	117.41 (10)
Li1—O6—H4A	121 (2)	C2—C3—N4	129.07 (10)
			
C1—N1—N2—C3	−0.25 (13)	N1—C1—C2—C3	−0.92 (14)
Li1—N1—N2—C3	169.92 (12)	N3—C1—C2—C3	175.50 (12)
C1—N1—N2—Li1^i^	−176.01 (12)	N1—C1—C2—C2^ii^	174.43 (9)
Li1—N1—N2—Li1^i^	−5.8 (2)	N3—C1—C2—C2^ii^	−9.15 (19)
Li1—O1—N3—O2	−173.04 (11)	N1—N2—C3—C2	−0.36 (14)
Li1—O1—N3—C1	4.91 (15)	Li1^i^—N2—C3—C2	175.68 (11)
Li1^i^—O3—N4—O4	−175.74 (11)	N1—N2—C3—N4	−179.10 (11)
Li1^i^—O3—N4—C3	4.81 (14)	Li1^i^—N2—C3—N4	−3.07 (17)
N2—N1—C1—C2	0.78 (14)	C1—C2—C3—N2	0.76 (14)
Li1—N1—C1—C2	−169.73 (11)	C2^ii^—C2—C3—N2	−174.58 (10)
N2—N1—C1—N3	−175.99 (10)	C1—C2—C3—N4	179.32 (13)
Li1—N1—C1—N3	13.49 (18)	C2^ii^—C2—C3—N4	4.0 (2)
O2—N3—C1—N1	166.52 (12)	O3—N4—C3—N2	−2.02 (17)
O1—N3—C1—N1	−11.53 (18)	O4—N4—C3—N2	178.50 (11)
O2—N3—C1—C2	−9.7 (2)	O3—N4—C3—C2	179.46 (12)
O1—N3—C1—C2	172.21 (13)	O4—N4—C3—C2	0.0 (2)

Symmetry codes: (i) −*x*+1, −*y*+1, −*z*; (ii) −*x*+1/2, −*y*+1/2, *z*.

### Hydrogen-bond geometry (Å, º)

**Table:**

*D*—H···*A*	*D*—H	H···*A*	*D*···*A*	*D*—H···*A*
O5—H1···O4^iii^	0.87 (2)	2.02 (2)	2.8818 (14)	174 (2)
O5—H2*A*···O2^iv^	0.87 (2)	2.13 (2)	3.0010 (15)	177 (4)
O5—H2*B*···O6^v^	0.87 (2)	2.02 (2)	2.8555 (17)	163 (3)
O6—H3···O1^vi^	0.82 (3)	2.24 (3)	2.9995 (17)	153 (2)
O6—H4*A*···O6^vii^	0.92 (4)	2.01 (4)	2.905 (2)	163 (4)
O6—H4*B*···O5^viii^	0.95 (5)	1.93 (5)	2.8555 (17)	164 (4)

Symmetry codes: (iii) −*x*+1/2, *y*, *z*+1/2; (iv) −*x*+1, *y*+1/2, −*z*+1/2; (v) −*x*+3/2, *y*, *z*+1/2; (vi) *x*, −*y*+1/2, *z*−1/2; (vii) −*x*+3/2, −*y*+1/2, *z*; (viii) −*x*+3/2, *y*, *z*−1/2.

## References

[bb1] Bauzá, A., Sharko, A. V., Senchyk, G. A., Rusanov, E. B., Frontera, A. & Domasevitch, K. V. (2017). *CrystEngComm*, **19**, 1933–1937.

[bb2] Brandenburg, K. (1999). *DIAMOND.* Crystal Impact GbR, Bonn, Germany.

[bb3] Domasevitch, K. V., Gospodinov, I., Krautscheid, H., Klapötke, T. M. & Stierstorfer, J. (2019). *New J. Chem.* **43**, 1305–1312.

[bb4] Domasevitch, K. V. & Ponomarova, V. V. (2021). *Acta Cryst.* E**77**, 1109–1115. 10.1107/S2056989021010227 PMC8587976 34868646

[bb5] Drukenmüller, I. E., Klapötke, T. M., Morgenstern, Y., Rusan, M. & Stierstorfer, J. (2014). *Z. Anorg. Allg. Chem.* **640**, 2139–2148.

[bb6] Dufter, A. M. W., Klapötke, T. M., Rusan, M., Schweiger, A. & Stierstorfer, J. (2020). *ChemPlusChem*, **85**, 2044–2050. 10.1002/cplu.202000427 32909700

[bb7] Dufter-Münster, A. M. W., Harter, A. G., Klapötke, T. M., Reinhardt, E., Römer, J. & Stierstorfer, J. (2022). *Eur. J. Inorg. Chem.* e202101048.

[bb8] Dunitz, J. D. (1995). *X-ray Analysis and the Structure of Organic Solids*, 2nd corrected reprint, pp. 106–111. Basel: Verlag Helvetica Chimica Acta.

[bb9] Farrugia, L. J. (2012). *J. Appl. Cryst.* **45**, 849–854.

[bb10] Glück, J., Klapötke, T. M., Rusan, M., Sabatini, J. J. & Stierstorfer, J. (2017). *Angew. Chem. Int. Ed.* **56**, 16507–16509. 10.1002/anie.201710746 29144054

[bb11] Gospodinov, I., Domasevitch, K. V., Unger, C. C., Klapötke, T. M. & Stierstorfer, J. (2020). *Cryst. Growth Des.* **20**, 755–764.

[bb12] Gural’skiy, I. A., Escudero, D., Frontera, A., Solntsev, P. V., Rusanov, E. B., Chernega, A. N., Krautscheid, H. & Domasevitch, K. V. (2009). *Dalton Trans.* pp. 2856–2864. 10.1039/b818125j 19333511

[bb13] Hirshfeld, F. L. (1977). *Theor. Chim. Acta*, **44**, 129–138.

[bb14] Janssen, J. W. A. M., Kruse, C. C., Koeners, H. J. & Habraken, C. (1973). *J. Heterocycl. Chem.* **10**, 1055–1058.

[bb15] McKinnon, J. J., Spackman, M. A. & Mitchell, A. S. (2004). *Acta Cryst.* B**60**, 627–668. 10.1107/S0108768104020300 15534375

[bb16] Olsher, U., Izatt, R. M., Bradshaw, J. S. & Dalley, N. K. (1991). *Chem. Rev.* **91**, 137–164.

[bb17] Ponomarova, V. V., Komarchuk, V. V., Boldog, I., Krautscheid, H. & Domasevitch, K. V. (2013). *CrystEngComm*, **15**, 8280–8287.

[bb18] Ravi, P. (2015). *J. Mol. Struct.* **1079**, 433–447.

[bb19] Rossi, M., Rickles, L. F. & Glusker, J. P. (1983). *Acta Cryst.* C**39**, 987–990.

[bb20] Sabatini, J. J. (2018). *Prop. Explos. Pyrotech.* **43**, 28–37.

[bb21] Shannon, R. D. (1976). *Acta Cryst.* A**32**, 751–767.

[bb22] Sheldrick, G. M. (2008). *Acta Cryst.* A**64**, 112–122. 10.1107/S0108767307043930 18156677

[bb23] Sheldrick, G. M. (2015). *Acta Cryst.* C**71**, 3–8.

[bb24] Spackman, M. A. & Byrom, P. G. A. (1997). *Chem. Phys. Lett.* **267**, 215–220.

[bb25] Spackman, M. A. & McKinnon, J. J. (2002). *CrystEngComm*, **4**, 378–392.

[bb27] Stoe & Cie (2000). *IPDS Software*. Stoe & Cie GmbH, Darmstadt, Germany.

[bb28] Turner, M. J., McKinnon, J. J., Wolff, S. K., Grimwood, D. J., Spackman, P. R., Jayatilaka, D. & Spackman, M. A. (2017). *CrystalExplorer17*. University of Western Australia. http://crystalexplorer.scb.uwa.edu.au/.

[bb29] Veluthaparambath, R. V. P., Krishna, V., Pancharatna, P. D. & Saha, B. K. (2023). *Cryst. Growth Des.* **23**, 442–449.

